# Dr. Ravi Nehru: The life, the man, the loss

**Published:** 2011

**Authors:** Amit Batla, Farah Khanam

**Affiliations:** Department of Neurology, All India Institute of Medical sciences, New Delhi, India; 1 Department of Neurology, G.B. Pant Hospital, New Delhi, India

**Figure:**
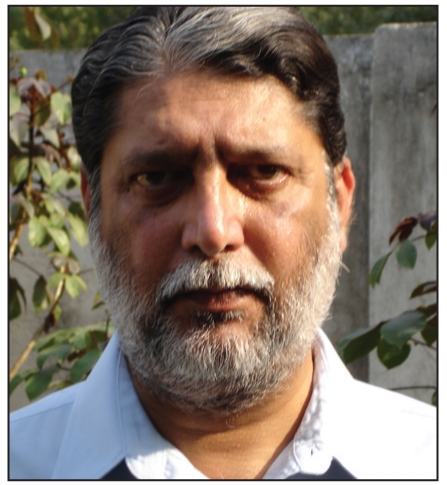
Dr. Ravi Nehru (30 July 1952–14 December 2010)

Dr. Ravi Nehru had been a wonderful teacher for his students, a caring doctor to his patients, an eminent neurologist, psychiatrist, neuropsychologist, and neurolinguist. No words can describe his aura; the ones who have felt it know that he was calm, poised, well mannered, loving, and being near him gave every one around him peace and sanity. It was often that a resident would come to him baffled with queries and issues. He would smile and politely ask him to sit down, have a coffee and suddenly all the queries would be settled by simple answers and all issues resolved without a doubt. He was admired by his colleagues for his helping nature, cool head and work ethics.

He was born on 30 July 1952. He spent his childhood in Meerut and Lucknow. He completed his MBBS from Maulana Azad Medical College (MAMC) in 1976. He always wanted to do MD Psychiatry, but at that time, the course did not exist; so, he did MD in Medicine in 1980. He went on to do D.M. Neurology from G.B. Pant hospital in 1984. He did not hesitate to pursue his inclination in psychiatry, and after D.M., he joined D.N.B. Psychiatry from G.B. Pant hospital and completed it successfully in 1992. This gave him the distinction of being the only professional in India formally trained and qualified in Medicine, Neurology and Psychiatry with two doctorates and one post-doctoral degree. If one would ask him about his knowledge, however, he would be happier talking more about his knowledge of Persian, Urdu, Arabic, Latin, Sanskrit and Spanish literature and language. He was studying Bengali in his last few days and had started reading Bengali literature. His knowledge of philosophy, spirituality and religions, though not certified, had been his most valuable asset. His personal library has more than a 5000 books on subjects that interested him including dog breeds, flowers, fishes and wines. He has been remembered in the autobiography of Khushwant Singh and a number of books have been presented to him by this eminent author.

He headed Neurobehaviour Clinic, Movement Disorders Clinic and Neurolinguistics Lab in Department of Neurology, G.B. Pant Hospital. He also taught and did research guidance at the post graduate and doctoral level, (M.D., D.M., M.Phil., Ph.D.) in Medicine, Neurology, Psychiatry, Neuropsychiatry, Behavioral Neurology, Neuropsychology and Neurolinguistics. He was engaged in teaching advanced courses at the University of Delhi as a visiting faculty in Neurolinguistics and Clinical Neuropsychology. He is renowned as the first neurolinguist in the country. He had been a member or advisor for several international and government bodies like Commission for Scientific and Technical Terminology, Expert Panel, Good Clinical Practice and Clinical Trials (The Drugs Controller General of India), Advisory Committee, Medical Television and World Health Review.

He has over 275 publications including books, editorials, articles, papers and abstracts in refereed journals and refereed conference proceedings. He had been actively involved in research and academic assignments with the Government of India. He served as the Executive Editor of the Indian Edition of the Archives of Neurology (American Medical Association) and Editor of the Delhi Psychiatry Bulletin. He had delivered over 100 invited lectures at national and international conferences, and at institutions including King’s College Hospital and the Institute of Psychiatry, University of London; University College London, University of London; School of Special Education, University of Birmingham, UK; and Cork University Hospital, University of Cork, Ireland.

His clinical work was mainly in the field of behavioral neurology and movement disorders. He did pioneering research in dementia, dyslexia, behavioral disorders in children, aphasias, neuropsychiatry and general neurology. His work on scientific organization of the Hindi alphabet and of phonemes and graphemes in the brain is path breaking. His work on language organization and dyslexia done 15 years back remains unsung due to untimely death of his research associate, Anju Garg, who worked with him on the subject.

He had a special attachment to his parent institute, MAMC. He played an elemental role in the Faculty Association of Maulana Azad Medical College and Associated L.N.J.P.N., G.B. Pant and Guru Nanak Eye Hospitals. He compiled the detailed history of MAMC in the form of an article (available online) and a book. This work had been very close to his heart and makes every MAMC alumni proud.

He spent his last few days fighting as his usual self against all odds. He was cheerful and loving till his last day and never forgot to bless his near and dear ones. His fond memory and wonderful way to live continues to inspire everyone. He is survived by his father, Mr. H. M. Nehru (86 years), and a younger brother, Rajiv Nehru, who is an eminent lawyer in Delhi.

The disciplines of Neurology, Psychology, Linguistics and Psychiatry will always miss him and be deprived of what all he could do further, had it not been for his untimely demise.

